# Understanding genetic variations associated with familial breast cancer

**DOI:** 10.1186/s12957-024-03553-9

**Published:** 2024-10-10

**Authors:** Manjusha Pal, Doutrina Das, Manoj Pandey

**Affiliations:** Department of Surgical Oncology, Institute of Medical Sciences, Banaras Hindu University, Varanasi, 221005 India

**Keywords:** Breast cancer, Familial breast cancer, *BRCA*, *ATM*, *TOX3*

## Abstract

**Background:**

Breast cancer is the most frequent cancer among women. Genetics are the main risk factor for breast cancer. Statistics show that 15–25% of breast cancers are inherited among those with cancer-prone relatives. *BRCA1*,* BRCA2*,* TP53*,* CDH1*,* PTEN*, and *STK11* are the most frequent genes for familial breast cancer, which occurs 80% of the time. In rare situations, moderate-penetrance gene mutations such *CHEK2*,* BRIP1*,* ATM*, and *PALB2* contribute 2–3%.

**Methods:**

A search of the PubMed database was carried out spanning from 2005 to July 2024, yielding a total of 768 articles that delve into the realm of familial breast cancer, concerning genes and genetic syndromes. After exclusion 150 articles were included in the final review.

**Results:**

We report on a set of 20 familial breast cancer -associated genes into high, moderate, and low penetrance levels. Additionally, 10 genetic disorders were found to be linked with familial breast cancer.

**Conclusion:**

Familial breast cancer has been linked to several genetic diseases and mutations, according to studies. Screening for genetic disorders is recommended by National Comprehensive Cancer Network recommendations. Evaluation of breast cancer candidate variations and risk loci may improve individual risk assessment. Only high- and moderate-risk gene variations have clinical guidelines, whereas low-risk gene variants require additional investigation. With increasing use of NGS technology, more linkage with rare genes is being discovered.

## Background

**Fig. 1:**
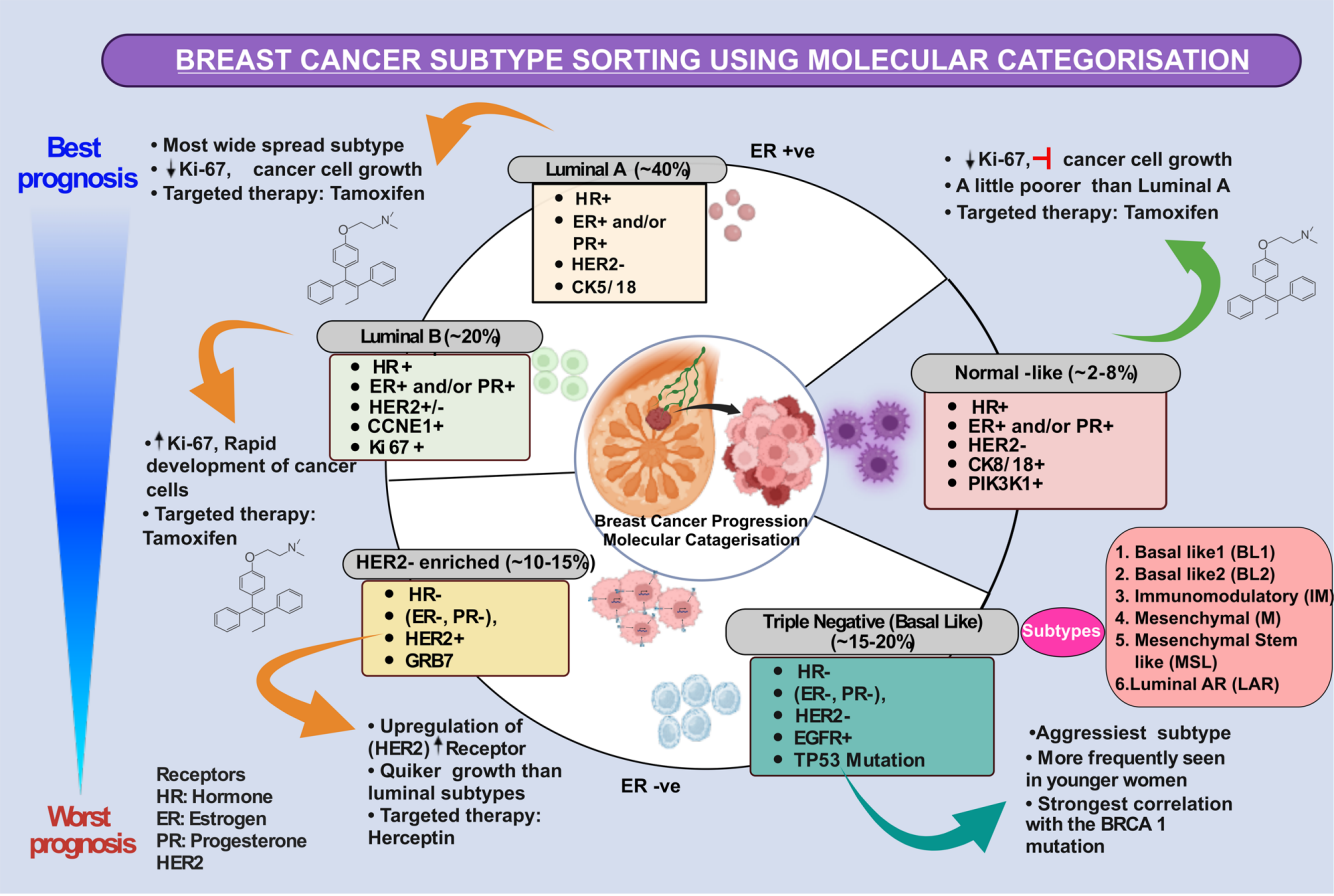
Breast cancer molecular categorization: The molecular classification, therapy, and prognosis. Hormone expression negatively impacts cell proliferation and tumor grade. The Luminal A subtype responds better to endocrine treatment, while TNBC is advanced, nuclear, and mitotically active and has a poor prognosis. (ER + and/or PR+ (Estrogen Receptor Positive and/or Progesterone Receptor Positive) HER2- (Human Epidermal Growth Factor Receptor 2 Negative), HR+ (Hormone Receptor Positive))

**Fig. 2:**
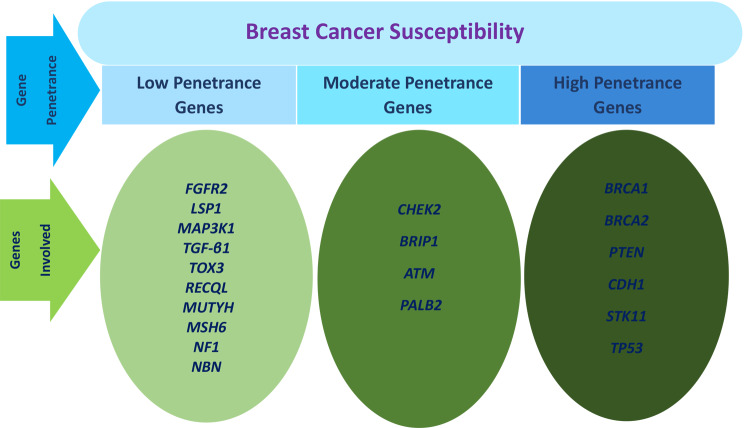
Classification of familial breast cancer genes based on their penetrance: Carrier mutations for genes predisposed to BC are classified into three penetrance levels: high, intermediate, and low

Breast cancer (BC) is the most common cancer among women. GLOBOCAN 2022 Statistics reveal that 2,296, 840 new cases of BC were identified globally, making it the fourth leading cause of cancer mortality [1]. Hereditary and environmental factors, including cell-cycle gene alterations, cause cancer. The abnormalities may be inherited, induced by carcinogens, age, hormonal variables, reproductive history, menstrual cycle, alcohol, radiation, and genetic susceptibility [2–5].BC classification considers treatment and prognostic factors, such as histopathological type, grade, stage, receptor status, and gene expression/mutation, as clinical and histopathologic factors (tumour size, lymph node involvement, metastasis) as histology alone fails to precisely predict tumour behaviour [6]. Extensive gene and protein expression profiling has identified four clinically significant molecular subtypes of BC [7]. Modern molecular pathology sought an explanation for BC heterogeneity using high-throughput biomarker screening. It offers biomarkers—ER (estrogen receptor), PR (progesterone receptor), and HER2 (human epidermal growth factor receptor 2) —that classify BC into five subtypes (Fig. 1): luminal A and B, HER2 enriched, triple-negative or basal-like (BL), and normal-like BC [8]. Beyond these molecular classifications, BC can be categorized epidemiologically into familial breast cancer (FBC), hereditary breast cancer (HBC), and sporadic breast cancer (SBC) [9, 10]. Approximately 15–25% of BC cases are hereditary, often occurring in women with affected first or second-degree relatives [11]. Additionally, cases of FBC in young adults are often inherited. Based on their penetrance the 3 categories and their corresponding genes are detailed (Fig. 2) [12–14]. Up to 25% of BC are associated with highly penetrant genes such as *BRCA1*, *BRCA2*, *TP53*, *CDH1*, *PTEN*, and *STK11*. Another 2-3% of cases result from rare, moderate-penetrance gene mutations like those in *CHEK2*, *BRIP1*, *ATM*, and *PALB2*, each doubling the risk [15, 16]. Although hereditary breast and ovarian cancer elevate BC risk, over half of the genetic susceptibility to BC remains unexplained. Given the considerable diversity among BC patients, the occurrence and genetic susceptibility of FBC vary based on race and geography. Genetic diseases such as hereditary breast and ovarian cancer (HBOC) syndrome are also associated with FBC. Li-Fraumeni syndrome mutations in TP53 boost cancer risk before age 30 and virtually assure cancer by age 60 [5]. *STK11*, *PTEN*, and *ATM* are implicated in syndromes like Peutz-Jeghers syndrome (PJS), Cowden syndrome (CS), and Ataxia–Telangiectasia (Louis-Bar Syndrome) respectively [17]. This study examines the prevalence of a family history of BC among women, delves into recent insights on FBC genetic susceptibility gene mutations, polymorphisms, and disease-related variations, and discusses recommendations for genetic counselling referrals and follow-up for gene mutation carrier.

## Methods

From searching the electronic database, 768 articles were identified. Research papers were sourced from PubMed, Medline and Scopus, spanning from 2005 to July 2024.The primary search terms employed were “familial breast cancer”, “family breast cancer”, and “gene,” confined to titles or abstracts. The subsequent criteria were used for selecting articles:

1. Articles involving patients who had a familial history of BC.
2. Articles that addressed the genetic predisposition concerning FBC.

**Fig. 3:**
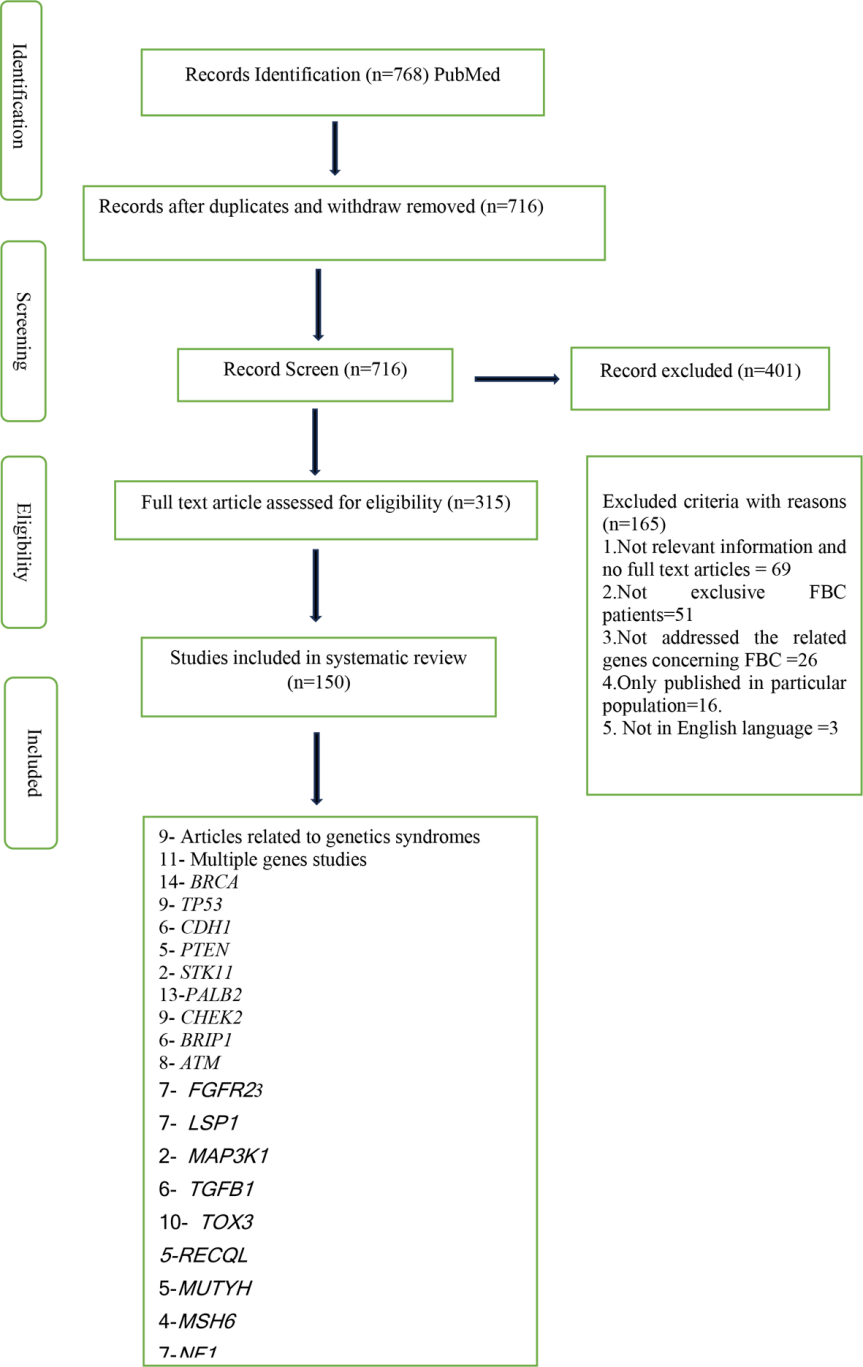
Flow diagram showing literature review and article selection

This study excluded studies featuring unrelated, as well as those lacking a cross-sectional design. The duplicates among databases, case reports, and non-English articles were excluded. Abstracts were read for all identified articles, and irrelevant articles were removed. After conducting a comprehensive review of the available literature, 69 articles were excluded due to either including irrelevant content or not being available in full-text format. 51 further studies were omitted because they did not only focus on patients with FBC. Furthermore, 26 studies were excluded due to their lack of emphasis on the specific genes linked to FBC. 16 studies were excluded because they focused on particular demographics, which limits the generalizability of their findings and 3 were not documented in English language. Finally, this analysis identified 150 FBC studies with 20 predominantly linked genes based on penetrance and 10 significant genetic diseases (Fig. 3).

## Results

Table 1 discusses the genetic vulnerability to FBC, including genes with additional gene characteristics related to different malignancies, localisation, syndrome, function, therapy, and prevention. High penetrance genes increase BC susceptibility due to mutations that significantly increase the likelihood of developing the disease over an individual’s lifetime. These genes may lead to a lifetime risk of BC as high as 80% [18]. Moderate-penetrance genes like *CHEK2*,* BRIP1*,* ABM*, and *PALB2* increase the likelihood of FBC by 20–50% throughout an individual’s life. High-penetrance genes in FBC are crucial for DNA repair and tumor suppression, increasing cancer risks when mutations occur. Moderate-penetrance genes have a lesser effect on risk and are more frequently mutated in the general population. Low-penetrance genes contribute to risk in a less obvious way, often requiring multiple variations to increase vulnerability. Understanding these differences is essential for genetic counselling and risk management in families with a history of BC. A range of genes, exhibiting high predisposition to intermediate and poor outcomes, have been linked to FBC (approximately 30%). Such instances are often observed in families with a high incidence of BC [19]. Notably, genes like *BRCA1/2* are connected to FBC, contributing to around 5% of BC-related mutations and potentially accounting for 16–25% of FBC cases [20, 21]. Additionally, mutations in genes such as *TP53*, *PTEN*, *STK11*, and *CDH1* are responsible for 5% of the risk associated with FBC and are linked to hereditary disorders. Low-sensitivity genes contribute to about 18% of the risk associated with FBC.

**Table 1:** **Breast cancer genes**: localization, syndrome, function, therapy, and prevention

Gene		Chromosome number	Syndrome	Others associated factor	Gene function	Treatment/prevention	References
Abbreviation	Expended form					Approved or in trial	
*BRCA1*	Breast Cancer gene 1	17q21	Hereditary Breast Ovarian Cancer syndrome	Ovarian, Pancreatic, Fallopian tube cancer	Involved in repairing damaged DNA in response to cellular stress	PARP Inhibitors	[156–158]
*BRCA2*	Breast cancer gene 2	13q12.3	Hereditary Breast Ovarian Cancer	Ovarian, Pancreatic, Prostate, Fallopian tube, Biliary cancer	Involved in repairing damaged DNA in response to cellular stress	PARP Inhibitors	[18, 159, 160, 157]
*TP53*	Tumor Protein 53	17p13.1	Li-Fraumeni syndrome	Childhood Sarcoma Brain Tumour, Adrenocortical carcinoma	Protection against replication of DNA damage	eprenetapopt, PRIMA-1, MET PEITC (phenethyl isothiocyanate) Ganetespib, TAS-102, Talazoparib Adavosertib	[159, 161]
*CDH1*	Cadherin-1 Homo sapiens	16q22.1	Hereditary lobular breast cancer syndrome	Ovarian, Endometrial carcinoma Gastric, Prostate cancer, colorectal	Cellular adhesion regulation Epithelial cell regulation	Surgery	[18, 162, 163]
*PTEN*	Phosphatase and TENsin homolog deleted on chromosome number 10	10q23	Cowden syndrome	Prostate, Thyroid, Endometrial cancer (PTEN Hamartoma Tumor Syndrome PHTS	Gatekeeper, cell cycle control, suppresses cell cycle progression and induction	PARP inhibitors Rigosertib mTOR inhibitors	[163–165, 18, 166]
*STK11*	Serine/threonine Kinase 11	19p13.3	Peutz-Jeghers syndrome	Pancreatic, Testicular, Colon, pancreas, ovarian sex cord-stromal tumors	Maintenance of energy homeostasis regulates members of the AMP-activated	Bemcentinib Talazoparib Sotorasib Adagrasib	[18, 167, 168]
*CHEK2*	Checkpoint Kinase 2	22q12.1	Li-Fraumeni syndrome	Prostate, Osteosarcoma, Lung, Colon, Kidney	A protein kinase has a role in cell cycle regulation at G2. Rapidly phosphorylation-activated CHEK2 interacts with BRCA1 and stabilizes	PARP inhibitors	[163, 169, 170]
*ATM*	Ataxia Telangiectasia mutated	11q23	Ataxia Telangiectasia	Ovarian, Prostate	Sensor in cellular response to DNA double strand breaks	PARP inhibitors ATM inhibitors	[101, 18]
*BRIP1*	BRCA1 interacting protein	17q23.2	Type J Fanconi anemia	Prostate, Brain, Cervix, Colon, kidney, Ovary, Pharynx, Skin, and vaginal	Engages the BRCA1 C –Terminus (BRCT) domain in interaction	PARP inhibitors	[18, 63, 171, 172]
*PALB2*	Partner and localizer of BRCA2	16P12.2	Fanconi anemia type N	Pancreatic, ovarian	Partners with BRCA2 involved in nuclear stability and localization	PARP inhibitors	[60, 101, 59]
*FGFR2*	Fibroblast growth factor receptor 2	10q26	-	Gastric, Lung, Ovarian, and Endometrial	Embryonic development and tissue regeneration, including bone and blood vessels	Pemigatinib Infigratinib Erdafitinib Derazantinib Non selective TKI	[94, 95, 173]
*LSP1*	Lymphocyte associated protein 1	11p15.5			Controls trans endothelial migration, adherence to fibrinogen matrix protein		[10, 103]
*MAP3K1*	Mitogen activated protein kinase 1	5q11.2	-	Prostate, Stomach, diffuse large B cell lymphoma	Regulates cell death, survival, migration, and differentiation	MEK inhibitors RAF inhibitors	[109, 115, 174]
*TGFB1*	Transforming growth factor beta 1	19q13.1	-	Colorectal, Lung, Liver, Prostate	Modulation of cellular development, differentiation, homeostasis, endothelial cell plasticity, immunoregulation, and apoptosis	Galunisertib Fresolimumab	[111, 115, 175, 176]
*TOX3*	TOX high mobility group box family member 3	*16q12.1*	-	Lung	Encodes a protein with an HMG box, which is thought to modify DNA and chromatin structure. Adversely controls BRCA1 expression by binding to the BRCA1 promoter		[177–179]
*RECQL*	RecQ like helicase	*12p12.1*	-	ovarian cancers, tongue, squamous cell carcinoma	DNA repair, including mismatch repair, nucleotide excision repair and direct repair	Topoisomerase I (TOP1) inhibitors. Better response to endocrine therapy	[180, 181]
*MUTYH*	mutY DNA glycosylase	*1p34.3–p32.1*	MUTYH polyposis syndrome	colorectal, Stomach, small intestine, ovary, endometrium, bladder	Involves the repair of DNA damaged by oxidative stress through base-excision repair during cell division	No specific therapies	[131, 134]
*MSH6*	MutS Homolog 6	*2*	Lynch syndrome	Colorectal, brain and blood cancer	DNA repair	Pembrolizumab and regorafenib	[135]
*NF1*	neurofibromin 1	*17*	Neurofibromatosis 1 Noonan syndrome	Sarcomas, brain, Ovarian and Melanoma cancer.	Regulates the RAS/MAPK and PI3K/mTOR Inhibits the activity of the Ras protein, which stimulates cellular proliferation and differentiation.	No specific therapies	[139, 140]
***NBN***	nibrin	*8*	Nijmegen Breakage Syndrome	Skin and Prostate cancer	Repair of damaged DNA.	PARP Inhibitors; platinum based chemotherapy; immunotherapy	[145, 147, 148]

### BRCA

*BRCA1* and *BRCA2* are tumor suppressor genes that repair DNA and regulate cell growth. BC risk increases considerably with gene mutations via autosomal dominant inheritance. *BRCA1*, on chromosome 17q21, encodes a 220 kDa nuclear phosphoprotein with 1863 amino acids in 24 exons [21]. *BRCA1* exons are divided into N-terminal RING fingerprint domain, C-terminal BRCT domain, each playing critical roles [22]. *BRCA2*, located on chromosome 13q12.3, encodes a 380 kDa protein with 27 distinct domains, including a transcriptional activation domain, a middle section with 8 BRC repeats binding to RAD51, a C-terminus DNA binding domain, nuclear localization signals, and a TR2 domain stabilizing RAD51-DNA interactions [23, 24]. *BRCA1/BRCA2* gene mutations account for 16-25% of FBC cases and 5% of BC-related gene mutations [25, 21] with *BRCA1*-linked tumors lacking ER expression and *BRCA2*-linked tumors showing ER positivity [22]. Over 2000 mutations have been reported in *BRCA1*/*BRCA2* genes, including deletions, insertions, and single nucleotide substitutions within coding or noncoding sequences [26]. The most common *BRCA1* variations are 185delAG, 5382insC, and C61G [27]. The 185delAG mutation, a recurring genetic alteration in the Southern Indian population, accounts for 24.6% of the disease-causing variations [28]. Common *BRCA2* mutations include 6174delT, 

10,204 A > T, 3036del4, and 6503delTT. In North-East Indian patients, 185DelAG, 1014DelGT, and 3889DelAG mutations in *BRCA1* exons 2 and 11 caused protein truncation [29]. Another common mutation, 3889DelAG, interacts with BRCA2 protein and is more common in the Northeast [29, 30]. *BRCA1* c.894delT, c.869delT, c.981–982delAT, c.1132delA, c.1252G > T, c.1953–1956delGAAA, c.5566 C > T, c.5533-5540delATTGGGCA, c.5154G > A, c.5215 + 2dupT, and in *BRCA2,* c.37G > T, c.262-263delCT, c.433dupG, c.439 C > T,

c.470–474delAGTCA, c.771–775delTCAAA, c.8377G > T, c.8584dupC, c.8687–8690delGTGC, c.10,150 C > T, c.7409dupT, c.7673–7674delAG, c.6547delG, c.7090G > T, c.3109 C > T are pathogenic or likely pathogenic variation reported in the Eastern Chinese population [31].

The *BRCA2* gene mutations c.3482dup and c.8878 C > T have been linked to an increased risk of BC in southern Brazil [32]. The variants c.5470_5477del and c.5521del in the *BRCA1* gene, as well as c.5167_5165del in the *BRCA2* gene, have been seen in Chinese descent [33, 34].

### TP53

*TP53*, also known as “the genome caretaker”, located on chromosome 17p13.1, encoding a 43.7 kDa phosphoprotein p53 with 393 amino acid residue [35]. The p53 polypeptide comprises various context-dependent functional domains, including the core DNA-binding domain, oligomerization domain, proline-rich domain, composite N-terminal transactivation domains (TAD1 and TAD2), and unstructured C-terminal domain (CTD) [36]. p53 mutations disrupt transcriptional processes, affecting DNA repair, senescence, apoptosis, autophagy, mitotic catastrophe, angiogenesis, and stress-induced phase transitions [37]. Notably, *TP53* mutations most frequently manifest in exons 5–8 and occur in approximately 30% of BC cases. Reports suggest that around 5% of BC patients had mutations in *CHEK2* or *TP53* when they possess a positive family history and wild-type *BRCA1/BRCA2* gene [38]. *TP53*’s proline-rich Pro72Leu/His/Arg (rs1042522) non-synonymous variation is remarkable. This exon 4 codon 72 polymorphism produces p53 proteins with different physicochemical and functional properties [39]. Recent studies show a strong relationship between the p53 codon 72 SNP with Indian vulnerability [40]. p.R337H Germline Variant among Women at Risk of HBC in a Public Health System of Midwest Brazil [41, 42],. According to the data, p. Arg181Cys is a founder pathogenic mutation that is most common among Arab Muslims in the Jerusalem and Hebron area [43].

### CDH1

*CDH1*, a tumor suppressor gene on chromosome 16q22.1, encodes a 120 kDa protein called E-cadherin [44]. It has 16 exons and 566 amino acids, with the C-Box motif in the N-terminal region influencing its connection with the APC/C complex. The cytoplasmic domain controls cellular functions like cell signalling, apoptosis, and invasion [45]. E-cadherin is a transmembrane protein essential for calcium-dependent cell-cell interaction, consisting of a transmembrane domain, a cytoplasmic domain, and five extracellular domains [44]. In the context of BC, E-cadherin’s normal activity serves as a deterrent against metastasis. However, *CDH1* mutations are connected to an aggressive BC pattern characterized by lymphovascular invasion and axillary lymph node metastases, particularly within Invasive Lobular Carcinoma (ILC), which accounts for 5–15% of BC cases and is linked to *CDH1* loss of function mutations [46, 47]. Individuals harbouring *CDH1* mutations face a lifetime risk of 39% for developing BC, with a strong association to LBC. Among the Pashtun ethnic population of Khyber Pakhtunkhwa, the *CDH1* (c.48 + 6 C > T, rs3743674) polymorphism has been identified as a contributing factor to an elevated risk of BC [48]. Furthermore, subsequent investigation unveiled the involvement of the *CDH1* 160 C/A (c.-124–161 C > A, rs16260) polymorphism in BC susceptibility [49].

### PTEN

Phosphatase and Tensin Homolog (*PTEN*), a BC-associated tumor suppressor gene on chromosome 10q23, is essential for survival and proliferation. It is 47.14 kDa and encodes 403 amino acids in 9 exons [50]. *PTEN* has N-terminal tyrosine phosphatase, C2-membrane binding, and PDZ-interaction motif domains. *PTEN*, a protein encoding phosphatidylinositol 3,4,5-triphosphate 3-phosphatase, is involved in the PI3K/AKT-mTOR signaling pathway, competing with PI3K and mitogen-activated protein kinase pathways to regulate cellular processes with lipid phosphatase activity [51]. Inactivation can occur through somatic mutations, gene deletions, and post-translational changes. Functional impairment from monoallelic or biallelic deletions and promoter methylation is common in *PTEN*. In 40–50% of BC, heterozygosity loss of *PTEN* gene occurs, with frameshift mutations being the main cause [52, 53].

In female Cowden Syndrome (CS) patients, the lifetime risk of BC ranges from 25 to 50%, and *PTEN* germline mutations are identified in 80–90% of CS families. Moreover, approximately 75% of female CS patients display various benign breast lesions such as fibroadenomas, cystic lesions, and ductal hyperplasia. The *PTEN* c.697 C > T (p. Arg233Ter, rs121909219) mutation introduces a premature stop codon in exon 7 of the gene encodes C2 domain and is linked to BC [54].

### STK11

Serine/threonine protein kinase 11, regulates the cell cycle, promotes apoptosis, and inhibits tumor growth. On chromosome 19p13.3, *STK11* has 9 coding and 1 non-coding exons. This 433-amino-acid, 50-kDa protein has an N-terminal kinase domain, a C-terminal regulatory domain [55]. *STK11* mutation carriers had a 32–54% probability of BC, rising from 8% at 40 to 32% at age 60 [10]. For patients diagnosed with PJS, the lifetime probability of developing BC ranges from 24 to 54%, typically manifesting around the age of 39 [56]. In the general population, a missense variant p.S422G has been identified in the *STK11* [57].

### PALB2

The Partner and Localizer of *BRCA2* gene (*PALB2*), is located on chromosome 16p12.2. The *PALB2* gene includes 13 exons, encoding a 1186 residues protein with 130 kDa size. *PALB2* possess a core chromatin-associated motif, a coiled-coil domain at the N terminus that interacts with *BRCA1*, and a WD40 repeat domain at the c terminus that binds *BRCA2*[58]. Bi-allelic *PALB2* germline mutations lead to Fanconi anaemia, whereas mono-allelic *PALB2* germline mutations elevate the risk of breast, pancreatic, and ovarian cancer [59–61]. New investigations showed germline *PALB2* mutations in BC families, indicating that *PALB2* could serve as an FBC tumor suppressor [62, 63]. The presence of *PALB2* mutations increases BC risk by 2–3 times, with carriers facing a cumulative risk of 35% within 0.6–2.7% of familial cases [64, 65].In Finland, a novel mutation (c.1592delT) led to a 4-fold increase in risk among individuals with or without a FH of the disease [63]. Various studies have indicated a modest risk associated with *PALB2* mutations, displaying moderate penetrance in fewer than 1% of unselected BC cases and less than 3% in individuals with a FH of BC. Research from the UK, Finland, Italy, Spain, and Canada shows that *PALB2* mutations are more prevalent in BC patients with a strong FH compared to unaffected controls [66]. Common SNPs within *PALB2* exons, such as c.2586 + 58C > T (rs249954), c.2997-624G > C (rs447529), and c.1684 + 1597T > C (rs16940342), have a strong association with susceptibility to BC [67]. In addition, recent studies have identified specific mutations like c.3114-1G > A (rs886039619) and c.1057 A > G (c.1057 A > G) as frequent in FBC cases [68]. A missense mutation, c.1676 A > G (rs152451), was discovered in 31.1% of 122 multi-ethnic Malaysian BC patients, including 82 Chinese, 25 Malaysian, 12 Indian, and 3 miscellaneous cases [69]. Similarly, a study on the *PALB2* gene within the North Indian population identified the mutation c.780delG in three patients with a high FBC risk, with a frequency of 1.5%. Furthermore, a novel mutation c.725delT was found in two patients with a frequency of 1% [70].

### CHEK2

The tumor suppressor gene *CHEK2* is located on chromosome 22q12.1 and it encodes a 65 kDa protein consisting of 543 amino acids. It plays a vital role in DNA repair, cell-cycle regulation, and the apoptotic response to DNA damage. *CHEK2*, N-terminal region contains a SQ/TQ cluster domain for phosphorylation in response to DNA damage, the fork head-associated protein interaction domain (FHA) for activation and rapid phosphorylation, and the C-terminal domain possesses serine/threonine kinase activity [71]. *CHEK2* mutations are rare, individuals carrying truncating mutations are more susceptible to developing BC. The risk is correlated with FH, rising notably when carriers have affected first and second-degree relatives [72]. In carriers lacking affected relatives, the risk stands at approximately 20%, while carriers with affected relatives may see the risk climb to 44% [73]. The protein-truncating variant 1100delC (p. Thr367fs, rs555607708r) raises BC risk by two to three times in general risk [74], with 0.2–1.6% of Northern and Eastern Europeans harbouring this mutation, known as *CHEK2* PV (Pathogenic variant) [75–77], while FBC cases were 4.8-fold [78].The 1100delC mutation has been specifically associated with ER-positive BC [74]. As a BC-sensitive factor, *CHEK2* is interconnected with DNA damage, replication checkpoint feedback, higher-grade malignancies, and bilateral disease [75].Czech individuals with FBC have a recurrent *CHEK2* gene variant, c.1009 − 118_1009-87delinsC, which disrupts pre-mRNA splicing and increases the risk of HBC [79].

### BRIP1

BRCA1-interacting protein 1, a DEAH helicase family member, is located on human chromosome 17q23.2 and consists of 20 exons encoding a protein with 1249 amino acids of 141 kDa weight. Its interaction with *BRCA1* is regulated by its N-terminal domain, playing a role in enhancing its DNA repair capabilities and tumor suppressor functions. Its C-terminal region has helicase activity and interacts with *BRCA1* via BRCT repeats [80]. Deficiency in *BRIP1* and constitutional truncating variants of *BRIP1* that elevate BC risk have been connected with Fanconi’s anemia [81]. *BRIP1* mutations contribute to about 1% of all BC [18]. The data indicates a significant correlation between two common polymorphisms, rs7220719 and rs11871753, and the risk of BC [82]. The Pro919Ser polymorphism (rs4986764), is strongly linked to BC susceptibility globally [83, 84]. However, a meta-analysis suggests that Asian women without *BRCA1/2* mutations and those with a FH of BC may be less likely to develop this polymorphism [85].

### ATM

Located on chromosome 11q23, the Ataxia-telangiectasia mutated (*ATM*) gene encodes a protein weighing 350 kDa, with 3056 amino acids, encoded by 66 exons on chromosome 11q23 [86]. *ATM*’s N-terminus contains multiple alpha helical repeat motifs and a critical region for interactions with proteins and DNA. It also has a FAT (FRAP) (FK506-binding protein 12-rapamcin-associated protein), ATM, TRAPP (Transformation/transcription domain-associated protein) domain and a FATC (FAT-C-terminal) domain on its C-terminal [87]. *ATM* serves as an intracellular sensor, activated in response to DNA double-strand breaks, and initiates phosphorylation of various downstream tumor suppressor proteins including *BRCA1*,* TP53*,* CHK2*, and *CHK1* [88]. *ATM* genes are linked to two- to four-fold a higher lifetime risk of breast cancer [89, 90].Moslemi et al. discovered that *ATM* missense mutations increase BC risk by a factor of 2.8 to 3.04 [91], with the c.7271T > G (rs28904921) missense mutation demonstrating the strongest association with BC [92, 93]. while the *ATM* p. Asp1853Val (rs1801673) missense variant exhibits the weakest correlation [86].

### FGFR2

Fibroblast growth factor receptor 2 (*FGFR2*) belongs to the family of tyrosine kinase receptors known as FGFR, which participate in various signalling pathways that impact cancer-related processes such as cell proliferation, apoptosis, and differentiation [94]. It is found on chromosome 10q26, encoding 22 exons, with 821 residues and molecular weight of 92.7 kDa. Overexpression of *FGFR2* is linked to 10–15% of BC [95, 96]. Genome-wide association studies have also identified SNPs within the second intron of the *FGFR2* gene as having a heightened association with an elevated risk of BC [97]. Further investigations have revealed that SNP within intron 2 of the *FGFR2* gene can alter the binding of transcription factors Oct-1/Runx2 and C/EBPb, leading to changes in *FGFR2* gene expression in breast tissue and cell lines [98]. The two intronic SNP variations of the *FGFR2* gene are rs1219648 and rs2981582, both located in intron 2 have been associated with BC [99, 100]. Another study linked the SNP rs1219648 with an increased risk of SBC in the North Indian population [96]. Furthermore, amplification of the chromosomal region of *FGFR1* (8p11-12) has been detected in approximately 10% of human BC, particularly those of the ER-positive subtype, and has been found to negatively impact overall survival [101].

### LSP1

Lymphocyte-specific protein 1 (*LSP1*) is a 339 amino acid F-actin binding protein found on chromosome 11p15.5, spans 20 exons, and has a molecular weight of 37.2 kDa [102]. It has an acidic N-terminal half and a basic C-terminal half. Its C-terminal half contains amino acid sequences homologous to the actin-binding domains of caldesmon and villin headpiece, making it a crucial F-actin binding protein [102]. *LSP1* plays a role in regulating neutrophil motility, the adhesion of fibrinogen matrix protein, and trans-endothelial movement [103, 104]. *LSP1* mutations has been identified in various conditions, including leukaemia, lymphomas, Hodgkin’s disease, and BC. The most prevalent alteration in the *LSP1* gene is the polymorphism rs3817198T > C, which has been extensively associated with an increased risk of BC [105–107]. These *LSP1* gene polymorphisms have the potential to modify protein expression, alter function, and impact downstream signalling pathways, ultimately influencing the risk of BC [16, 99, 108].

### MAP3K1

Mitogen activated protein kinase 1 is a serine/threonine kinase involved in the MAPK signalling cascade, located on chromosome 5q11.2. It has 20 exons, encoding 1512 residue protein of 196 kDa. It contains a plant homeodomain in its N-terminus and a phospho-kinase activity in its C-terminus. Numerous studies have demonstrated the involvement of *MAP3K1* in processes such as cell survival, apoptosis, and cell motility across various normal and malignant cell types [109]. One specific polymorphism of *MAP3K1*, rs889312 (rs889312 A > C), has been associated with an elevated risk of distant metastatic development in BC. The *MAP3K1* rs889312 polymorphism is linked to a higher risk of distant metastasis in BC with a mechanistic relationship identified in the Pakistani population, with the disease association strength being extensive in populations from East Asia, North Africa, and the Northern Hemisphere [110].

### TGFB1

TGF (transforming growth factor beta) is a pleiotropic growth factor that regulates cell survival, proliferation, apoptosis, and differentiation in a cell- and context-dependent way. TGF1, TGF2, and TGF3 are members of the TGF subfamily of cytokines. The *TGFB1* gene, a member of the TGFβ family, which is found on Chromosome 19q13.1, has 7 exons, encoding a protein of ~ 25 kDa [111].*TGFB1* is a 390 amino acid protein consisting of an N-terminal signal peptide, a pro-region called latency-associated peptide, and a C-terminal region that becomes the mature TGFβ molecule after proteolytic cleavage from the pro-region. Several analyses have shown that *TGFB1* has a dual effect on carcinogenesis, acting as a tumor suppressor in the early stages and a tumor promoter and metastasis propagator in the later stages of BC [112–114]. SNPs in the *TGFB1* genes rs1800468, rs1800469, rs1800470, and rs1800471 have been linked to BC susceptibility in several studies [115, 116]. A polymorphism in the *TGFB1* gene, specific thymine to cytosine transition in the 29th nucleotide in the coding sequence rs1982073 (29 C > T, p. Pro10Leu) has been linked to increased serum *TGFB1* levels and a increased likelihood of BC.

### TOX3

Located on 16q12.1 chromosome, the *TOX3* encodes the 576 amino acid nucleoprotein TOX High Mobility Group Box Family Member 3, with a 63.3 kDa molecular mass. It contains 7 exons, a nuclear localization signal in its N-terminal domain, an HMG box domain for DNA structural modification, and a polyglutamine stretch at its C-terminus [117, 118]. Clinical reports have indicated that individuals with elevated *TOX3* mRNA expression levels experience shorter overall survival, and a positive association has been established between higher *TOX3* mRNA expression and metastatic BC [117]. Studies by Riaz et al. have connected risk alleles rs3803662 and rs12443621 to lower *TOX3* mRNA expression, suggesting a potential tumor suppressor role for *TOX3* [119]. Moreover, susceptibility loci within *TOX3* have been linked to ER-positive BC verses ER-negative [120, 121]. It has been shown that *TOX3* activates ER and Bcl-2-sensitive promoters and modulates BRCA1 promoter expression [122–124]. *TOX3* plays a crucial role in cell proliferation, migration, and survival in response to apoptotic signals [82]. The SNP rs3803662:C > T is the most common genetic variant of *TOX3*, linked to BC and its T allele, which influences BC prognosis, advanced tumor stages, poor survival, and luminal molecular subtypes or ER-positive expression [125, 120].

### RECQL

*RECQL* is a member of the RecQ helicase protein family and is found on chromosome 12p12 and encodes a protein of 649 amino-acids [126]. It encodes DNA helicases and has a crucial function in ensuring the integrity of the genome. The prevalence of *RECQL* mutations in FBC patients is 2.0%, compared to 0.54% in the general BC population. Data reported that nonsense variant of *RECQL* at nucleotide position 225 in exon 4 (c.225G > A (p.W75*) was found in Pakistani population which is likely to cause the protein to end prematurely [127]. c.643 C > T;p.Arg215* was reported in French-Canadian women and c.1667_1667 + 3delAGTA was found in Polish women [128]. Data reported 16 mutation sites that are c.2 T > C, c.1805 C > T, c.1063 A > G, c.199G > A, c.1088 A > G, c.644G > A, c.631 A > G, c.1114G > A, c.1361G > A, c.1637 T > C, c.1090G > A, c.1123G > T, c.1211G > C, c.1382 A > G, c.700 + 1G > T, and c.1729 A > C found in Chinese patients [129].The use of *RECQL* mutations as a biomarker for pre-onset counselling is controversial because mutations are rare and the limited research on their relationship with clinical correlation and pathological features, especially in Asian populations [129].

### MUTYH

*MUTYH*, found on chromosome 1p34.3–p32.1, has 16 exons covering 1.65 kb. It produces a protein that protects DNA from harmful effects of cellular metabolism by eliminating modified bases. It functions as a tumor suppressor and operates recessively, requiring biallelic or homozygous mutations to disable its function [130] Autosomal recessive familial adenomatous polyposis 2 (FAP2) is a condition, an individual inherits two different versions of the *MUTYH* gene, which is responsible for the base excision repair (BER) process [131].Two primary genetic alterations, p.Tyr179Cys (p.Y179C) and p.Gly396Asp (p.G396D), were identified in the Non-Hispanic white population [132].Another genetic mutation, namely c.1187G > A, was discovered an Egyptian [133]. Studies suggest a slight link between breast cancer and monoallelic *MUTYH* mutations in Sephardi Jewish and Chinese women, but no significant risk was found in Canadian and Dutch cohorts [134].

### MSH6

The *MSH6* genes are crucial for DNA mismatch repair (MMR), and located on chromosome number 2. Lynch syndrome (LS) may result from these genes, with MMR gene mutations being prevalent in BC patients, according to studies, Lynch syndrome (LS) might arise from mutation in *MSH6* and common in BC patients [135]. The c.3013 C > T (p.Arg1005) mutation in the *MSH6* was found in the Chinese population [136], c.738 741delAAAA was found in Spain [137], c.3312delT mutation in the *MSH6* was found in Egyptian Study [133]. The clinical study investigating the association between LS and BC risk yields conflicting results, as several studies demonstrate a 2 to 3- fold rise in risk, while others fail to identify any indication of heightened risk [138].

### NF1

The *NF1* with a coding sequence of 8,517 base pairs, encodes a 2,839 amino acid protein with molecular mass of 319 kDa. Pathogenic variants of the *NF1* are the cause of neurofibromatosis 1, an autosomal dominant condition [139]. It inhibits tumor growth by regulating the activity of Ras guanosine triphosphatase, preventing GTPase activation, and regulating cell proliferation and differentiation [140].The study found that women with *NF1* who are 50 years or older have a lower risk of BC compared to the general population [139]. The *NF1* has a nonstop mutation, c.1915C > T; p.*639Arg, which results in the deletion of the stop codon, causing normal translation failure and potentially causing continuous translation of the downstream messenger RNA in the 3’ untranslated region [141]. This variant was observed in Latin American population, it is less frequent in Africa and more frequent in Europe [142]. The novel variant c.7000–2dupA was reported in the Turkish population [143]. Women diagnosed with *NF1* have been seen to have a 5-fold higher likelihood of developing BC, especially before the age of 50, in comparison to the overall population [144].

### NBN

*NBN* (Nibrin) is a protein encoded by the gene *NBS1* or Cell cycle regulating Protein P95, located on chromosome 8. It is part of the MRN/NMR complex, also known as the Double strand DNA break complex, which regulates cellular responses to DNA breakage and maintains chromosomal stability [145]. Women who have changes in the *NBN* gene, which codes for Nijmegen Breakage Syndrome (NBS), may be more likely to get BC [146]. *NBN* may lead to a higher risk that is around 2 to 3 times greater [147].The c.657_661del5 mutation is linked to increased BC risk, especially in individuals of Slavic and Eastern European descent [148]. The mutation c.-242-110delAGTA was found to be linked to a higher risk of getting BC in French Canadian families [149].

## Discussion

FBC constitute an inherited element within the spectrum of BC cases, contributing to approximately 5-7% of all instances of BC [16]. It’s estimated that a substantial 73% of the risk associated with developing BC within a family can be attributed to genetics, leaving the remaining 27% linked to environmental factors [5]. Despite the influence of hormone-related and lifestyle factors on increasing susceptibility, genetics remains the primary and most influential risk factor for the occurrence of BC. The earliest documented reference to FBC dates back to 1866 when Broca, recorded the history of BC in his wife’s family. Subsequently, in 1979, Lynch introduced the criteria that define FBC [150]. These defining features encompass an earlier age of onset, an elevated occurrence of bilateral and multicentric disease, and a family lineage featuring BC in two or more first-degree relatives.

As of now, a universally accepted definition for “familial” BC is lacking. However, specific indicators suggest the presence of FBC [151].

- BC in close relatives, with at least one case diagnosed before the age of 50.
- BC cases within family diagnosed Soon after turning 40.
- Male BC cases with a family history of ovarian cancer or early-onset female BC.
- Ashkenazi Jewish ancestry associated with BC; particularly TNBC (triple negative breast cancer) diagnosed before age 60.
- BC cases within family encompassing at least three instances of breast and/or ovarian cancer.

The literature review highlights the significant role of genetics in FBC, focusing on high-penetrance genes, moderate as well as low-penetrance genes. The review screened 768 studies from major databases, narrowing down to 150 studies that met stringent criteria. The identification of 20 key genes associated with FBC, and categorizing them based on penetrance, and emphasizes the complexity of genetic factors in BC risk. High-penetrance genes, such as *BRCA1* and *BRCA2*, are well-established as major contributors to FBC, often necessitating preventive measures. Moderate-penetrance genes, like *CHEK2*,* PALB2*, and *ATM*, elevate the risk but to a lesser extent than high-penetrance mutations. These findings reinforce the need for personalized approaches in genetic counselling and risk assessment. The review also identified 10 significant genetic syndromes associated with FBC, including hereditary breast and ovarian cancer (HBOC) syndrome, Li-Fraumeni syndrome, and Cowden syndrome, providing crucial insight into the broader implications of FBC. Understanding these syndromes is vital for clinicians to recommend appropriate surveillance and management strategies for affected families. The findings suggest the importance of integrating genetic testing into clinical practice, particularly for individuals with a family history of BC. Genetic testing for FBC significantly impacts screening, treatment, and counselling for at-risk individuals. The latest recommendations emphasize the importance of genetic testing and screening in cases of FBC: Priority should be given to individuals with a significant family history of BC, especially if numerous family members are affected. Personal history of cancer, particularly those diagnosed before the age of 50 or with certain tumor features, should also be assessed for genetic testing. The Gail and Tyrer-Cuzick models are useful for assessing individual risk based on family history and medical history [152], however their utility is limited to certain populations. The test plan should start with a family member experiencing symptoms to identify genetic variants, which can then be used to test non-symptomatic family members, enhancing the likelihood of detecting pathogenic variations. Organizations like the National Comprehensive Cancer Network provide comprehensive guidance on genetic testing and screening procedures for those at high risk [10]. Genetic testing has significant therapeutic consequences, enabling tailored risk management and preventative treatments. Clinical practice recommendations for FBC focus on identifying and treating hereditary cancer syndromes linked to pathogenic or probable pathogenic genetic variations, prioritizing genetic counselling, risk assessment, and management options for individuals with certain genetic variations. Despite the current limitations of multigene panel testing for practical use, it has the potential to become a significant tool in future FBC screening efforts. The BRCAPRO model uses Bayes theorem to predict BC, considering family prevalence and disease emergence age. The Myriad Model predicts mutation carriage, while the BOADICEA model considers simultaneous effects of BRCA1 and BRCA2, genetic modifiers, and low penetrance genes on BC risk. Breast imaging is used to evaluate women with breast complaints or clinical issues, while ultrasound is a portable tool for assessing breast masses [153]. Three primary methods for tissue sampling of mass or abnormality detected by physical examination or imaging are fine needle aspiration (FNA), core biopsy, and excisional biopsy, each with varying sensitivity, specificity, positive and negative predictive value, and training and health system requirements [153]. BC prevention faces numerous challenges, including identifying medications that reduce aggressive breast tumors, such as TNBC, HER2+, or luminal B subtypes. Tamoxifen and Raloxifene help reduce BC risk in women with higher risk due to hereditary factors, while postmenopausal women may be eligible for aromatase inhibitor treatment [154]. Surgical prophylaxis, particularly prophylactic bilateral mastectomy, is effective in preventing BC and reducing mortality in about 3% of women with a hereditary BRCA1/2 gene mutation [155]. Nipple-sparing mastectomies are a safe option for these women, but they may face additional challenges, such as psychological discomfort and resource concerns, due to changes in physical appearance [154].

## Conclusions

BC is a prevalent concern for women worldwide. To enhance BC prevention and treatment efforts, future risk assessment strategies may amalgamate high, moderate, and low penetrance genes. This review delves into mutations across these penetrance levels, illuminating familial BC predisposition. This comprehensive approach is pivotal for unravelling BC’s origins, refining diagnostics, and tailoring treatments. Exploring the entire genetic landscape promises a holistic comprehension of disease progression, offering novel avenues for diagnostic markers and targeted BC therapies. Highly penetrant gene mutations primarily contribute to BC cases, warranting specific guidelines for managing such patients. Moderate-penetrance mutations play a role in some cases, while low-penetrance alleles have surfaced through genetic testing. Although mutation testing necessitates suspicion of certain causes, next-generation sequencing holds promise for improved gene identification and clinical interventions.

Timely identification, treatment, monitoring, and survivorship care are crucial for the survival of individuals with breast cancer, potentially leading to significant reductions in mortality rates. Early diagnosis and screening are two interconnected strategies aimed at facilitating the prompt detection of cancer. Further research is needed to identify breast cancer-prone genes using whole-genome sequencing and Genome-wide association studies (GWAS). Future research should identify treatment targets and integrate genetic markers with clinical factors for better risk categorization and screening. Advances in genetic testing and gene editing could lead to personalized and more effective medicines.

## Data Availability

No datasets were generated or analysed during the current study.
